# Supplementary material for: External validity of prevalence estimates from the national maternity surveys in England: The impact of response rate
Source: PLoS One. 2020 Nov 30;15(11):e0242815. doi: 10.1371/journal.pone.0242815 (PMC7703875; doi:10.1371/journal.pone.0242815)
Supplement: Attachment — Submitted filename: Response to reviewers.docx [file pone.0242815.s001.docx]

Please find below our response to each point raised by the editor and reviewers.

Academic Editor

*Please ensure that your manuscript meets PLOS ONE's style requirements, including those for file naming.*

The manuscript has been revised in line with PLOS ONE’s style requirements using the templates provided. The references and the file names of the manuscript and the figures have also been updated.

*In your Methods section, please provide a link for every data source used.*

References to each of the reports for the previous national maternity surveys in 2006, 2010, 2014 and 2018 have been added to the methods section and the references section has been updated accordingly.

Reviewer 1

*First, the authors should cite more thoroughly the small number of previous publications exploring the impact of response rates on survey accuracy, including Curtin et al. (POQ), Keeter et al. (POQ), Merkle and Edelman (book chapter), Holbrook et al. (book chapter).*

We thank the reviewer for the positive review and for the suggestions for additional references. The manuscript has been revised and all of the above publications are now cited.

*Second, and more serious, I appreciate the authors' discussion of breastfeeding, but there's a problem here. Treating unknown breastfeeding status as not breastfeeding no doubt (as the authors say) biases those numbers downward from the routine data. And unlike the other measures, I presume that even the routine data for breastfeeding are based on parent reports rather than more objective data. So I would strongly suggest a different approach to breastfeeding: (1) be much more explicit with readers about how the routine data measurements were made, (2) tell readers the proportions of babies for whom breastfeeding was not known in each survey, (3) if those numbers are large enough, consider dropping the breastfeeding data from the paper altogether, (4) if those data are not dropped, discuss them completely separately from all other data, making it much clearer to readers that the routine data on breastfeeding may be problematic enough to undermine the value of the analyses with that variable*.

Thank you to the reviewer for raising this important point and for the helpful suggestions about how to approach the issue. We have made the following revisions:

- We have stated clearly how the prevalence of breastfeeding initiation status is measured in the routine data (page 8, lines 202-212 on revised manuscript with track changes).
- We have examined the proportion of women with missing data for each of the maternity indicators assessed in the surveys.
- The proportion of women with unknown breastfeeding initiation status was low across surveys (<4.3%) and so we have decided to include the survey data on this indicator in the analysis. However, the proportion of women with unknown breastfeeding status at 6-8 weeks was high, at least in the 2014 survey (25.0%). Therefore, in line with the reviewer’s recommendation, we have decided to exclude the breastfeeding status at 6-8 weeks indicator from the analysis.
- We have stated the proportion of missing data for each of the indicators across all of the surveys and for the routine data (page 15, table 4 on revised manuscript with track changes). This is 5% or less for all indicators.
- We have revised the discussion to make clearer the limitations with the routine data on breastfeeding initiation status and the potential impact of this on the validity of the analysis of the breastfeeding data (page 19, lines 430-432 on revised manuscript with track changes).

Reviewer 2

We thank the reviewer for the positive review and note there are no points to which we need to respond.
